# Whole-genome association analysis of pork meat pH revealed three significant regions and several potential genes in Finnish Yorkshire pigs

**DOI:** 10.1186/s12863-017-0482-x

**Published:** 2017-02-13

**Authors:** Lucas L. Verardo, Marja-Liisa Sevón-Aimonen, Timo Serenius, Ville Hietakangas, Pekka Uimari

**Affiliations:** 1 Department of Animal Science/Animal Breeding, Federal University of Viçosa, Viçosa, Brazil; 2 Green technology, Natural Resources Institute Finland, Jokioinen, Finland; 3 Figen Oy, Seinäjoki, Finland; 4 Department of Biosciences, University of Helsinki, Helsinki, Finland; 5 Institute of Biotechnology, University of Helsinki, Helsinki, Finland; 6 Department of Agricultural Sciences, University of Helsinki, Helsinki, Finland

**Keywords:** Association analysis, Meat quality, Pig, Post-GWAS analysis

## Abstract

**Background:**

One of the most commonly used quality measurements of pork is pH measured 24 h after slaughter. The most probable mode of inheritance for this trait is oligogenic with several known major genes, such as *PRKAG3*. In this study, we used whole-genome SNP genotypes of over 700 AI boars; after a quality check, 42,385 SNPs remained for association analysis. All the boars were purebred Finnish Yorkshire. To account for relatedness of the animals, a pedigree-based relationship matrix was used in a mixed linear model to test the effect of SNPs on pH measured from loin. A bioinformatics analysis was performed to identify the most promising genes in the significant regions related to meat quality.

**Results:**

Genome-wide association study (GWAS) revealed three significant chromosomal regions: one on chromosome 3 (39.9 Mb–40.1 Mb) and two on chromosome 15 (58.5 Mb–60.5 Mb and 132 Mb–135 Mb including *PRKAG3*). A conditional analysis with a significant SNP in the *PRKAG3* region, MARC0083357, as a covariate in the model retained the significant SNPs on chromosome 3. Even though linkage disequilibrium was relatively high over a long distance between MARC0083357 and other significant SNPs on chromosome 15, some SNPs retained their significance in the conditional analysis, even in the vicinity of *PRKAG3*. The significant regions harbored several genes, including two genes involved in cyclic AMP (cAMP) signaling: *ADCY9* and *CREBBP*. Based on functional and transcription factor-gene networks, the most promising candidate genes for meat pH are *ADCY9, CREBBP, TRAP1, NRG1, PRKAG3*, *VIL1, TNS1*, and *IGFBP5*, and the key transcription factors related to these genes are *HNF4A*, *PPARG*, and *Nkx2-5*.

**Conclusions:**

Based on SNP association, pathway, and transcription factor analysis, we were able to identify several genes with potential to control muscle cell homeostasis and meat quality. The associated SNPs can be used in selection for better pork. We also showed that post-GWAS analysis reveals important information about the genes’ potential role on meat quality. The gained information can be used in later functional studies.

## Background

The most common quality measurements of pork are pH and color. Both depend on post-mortem biochemical processes where lactate is produced from glycogen through glycogenolysis and anaerobic glycolysis, consequently lowering the pH and affecting the color of meat. The degree of glycolysis depends on the glycolytic potential, i.e. the amount of lactate that can be produced from glycogen in muscle at the time of slaughter [1]. Differences in glycolytic potential are known to be partly genetic. A well-known gene affecting meat quality is *PRKAG3* (protein kinase, AMP-activated, gamma 3 non-catalytic subunit) [2], formerly known as the *RN* gene [3, 4]. An R200Q substitution in this gene is related to the upregulation of certain key enzymes (e.g. UDP-glucose pyrophosphorylase) that increase glycogen production in the muscle cells of 200Q animals [5]. Additionally, several other genes are associated with post-mortem pH and color of pork loin and ham, including *RYR1* [6], *CAST* [7], and *PHKG1* [8] among others.

Genome-wide association study (GWAS), based on abundant SNP markers, is an effective tool to find chromosomal regions that explain at least a moderate proportion of the genetic variance of the studied trait in a certain population. Since the launch of the commercial SNP chip for pigs (Illumina PorcineSNP60 Genotyping Beadchip), several GWAS for pork meat quality traits have been published [9–15]. These studies have detected significant associations between SNPs and pH on the following chromosomes: chromosome 14 [9]; 2, 3, 4, 13, and X [10]; 1, 2, 3, 7, 9, and 13 [11]; 2 and 15 [12]; 1 and 8 [13]; and 15 [14, 15].

The effective population size (N_e_) of commercial pork breeds is relatively small; e.g. in Finnish Yorkshire, the pedigree- and marker-based estimate of N_e_ is only 60 animals [16]. This leads to strong linkage disequilibrium (LD) over long distances, and ultimately to a large number of genes surrounding the most significant SNP depending, of course, on how rich in genes the region is. Post-GWAS analysis, such as pathway and gene-transcription factor (TF) network analysis facilitate the identification and *in silico* validation of the most probable group of candidate genes in these regions and increases our understanding of the molecular mechanisms of the studied trait [17, 18].

In this article, we present the results from GWAS of loin pH measured 24 h post mortem using the estimated breeding values of Finnish Yorkshire boars. We also conducted a post-GWAS bioinformatics analysis of the significant genomic regions and genes.

## Methods

### Animals and phenotypes

The animals used in this study were AI boars of Finnish Yorkshire origin. The studied trait, pH of loin, was measured at the slaughterhouse 24 h post mortem using a Knick 752 pH meter and an Ingold 460 electrode (see Sevón-Aimonen et al. [19] for more information). The slaughtered pigs were grown in a test station. The animals arrived at the test station at 30 kg live weight and were sent for slaughter after 13 weeks of testing, at approximately 100 kg live weight. Prior to 2006, all pigs had restricted feeding based on their body weight; after 2006, feeding was close to *ad libitum*. The average pH for Finnish Yorkshire was 5.53 (SD = 0.15), based on 45,639 loin pH observations.

Estimated breeding values (EBVs) for the AI boars were calculated by Figen Oy (Seinäjoki, Finland) using a single-trait animal model and the Mix99 program package (MiX99, http://www.luke.fi/mix99. EBVs were mainly based on phenotypic measurements of half-sibs, full-sibs, and progeny. No phenotypic measurements of loin pH of the AI boars themselves were available. The statistical model included gender, slaughter batch, and time from slaughter to pH measurement as fixed effects, and litter and animal as random effects. Approximate reliabilities were calculated using ApaX, which is part of the MiX99 program package. All animals were related including, e.g., sire-son and half-sib pairs. Prior to association analysis, raw EBVs were deregressed and their weights calculated based on the method presented by Garrick et al. [20]. Deregressed EBVs (dEBVs) with weight less than 1.0 were removed from the data. The average reliability of the original EBVs was 0.56. The average dEBV was 0.09 and the SD 0.07, and the mean weight of the dEBVs was 3.6.

### Genotypes

DNA was extracted from the boars either from hair follicles (boars born before 2008) or semen (boars born after 2008). Whole-genome SNP genotyping was done at FIMM (Institute for Molecular Medicine Finland, Helsinki) or at GeneSeek (Lincoln, NE, USA) using the Illumina PorcineSNP60 BeadChip (Illumina Inc., San Diego, CA, USA). Only animals with call rate (CR) >0.9 were included into the statistical analysis, 703 animals in total. Additionally, SNPs with CR <0.9, minor allele frequency (MAF) <0.1, and *P*-value of Hardy-Weinberg test statistics <0.00001 were removed from the data prior to the statistical analysis. The final data included 42,385 SNPs. The map positions of the SNPs were based on the genome build Sscrofa10.2.

### Association analysis

Associations between loin pH and SNPs were tested for each SNP separately. Because the animals were related, the following linear model was applied to the data:

$$ {y}_i=\mu + b*{x}_i + {a}_i + {e}_i, $$

where *y*_*i*_ is the deregressed EBV of an AI-boar *i*; *μ* is an intercept; *b* is the regression coefficient; and *x*_*i*_ is the corresponding allele dose of the tested SNP with values of 0 (homozygous for the major allele), 1 (heterozygote), and 2 (homozygous for the minor allele); *a*_*i*_ is the polygenic effect with *a*_*i*_ ~ N(0, **A**σ^2^_a_); and *e*_*i*_ is the residual effect with *e*_*i*_ ~ N(0, **I**σ^2^_e_/*w*_*i*_). Matrix **A** is the pedigree-based additive relationship matrix and **I** is the identity matrix with diagonal elements of σ^2^_e_/*w*_*i*_, where weights (*w*_*i*_) were calculated based on the reliabilities of the EBVs of the animal *i* itself and its parents. The SNP effect (*b*) and additive genetic (σ^2^_a_) and residual (σ^2^_e_) variances were estimated for each SNP separately using the Restricted Maximum Likelihood (REML) method of the DMU program package [21].

The statistical significance of the SNPs was based on a two-sided *t*-test with H_0_: *b* = 0, H_A_: *b* ≠ 0, and Bonferroni-corrected *P*-values. Because of the strong LD between the SNPs in Finnish Yorkshire [16], the number of independent tests is less than the number of SNPs in the data. Thus, in this research we used the previously defined *P*-value of 2.0E-06 based on the assumption of 25,000 independent tests, as a limit value for statistically significant association [22]. Haplotypes of the animals were estimated with FastPHASE [23] using default parameters, and Manhattan plots were produced with the Haploview program [24].

### Post-GWAS analysis

For post-GWAS analysis, three lists of genes were formed based on genes within the significant regions: on chromosome 3 (39.4 Mb–40.1 Mb) and on chromosome 15 (58.5 Mb–60.5 Mb and 133.3 Mb–134.2 Mb). Additionally, we formed a separate list of genes relating to the significant SNPs on chromosome 15 outside the above regions, using 22.2 kb of the 5′ and 3′ flanking sequences (i.e. half the average distance between two SNPs on the chip). The annotations were based on the genome build Sscrofa10.2 at the NCBI website (https://www.ncbi.nlm.nih.gov/gene/). The two-sided hypergeometric test of the ClueGO plug-in of Cytoscape [25] was used to construct a gene network highlighting the pathways and relations across the four sets of genes.

The TFM-Explorer program (http://bioinfo.lifl.fr/TFM/TFME/) was used to identify the TF related to significant SNPs and regions. The TFM-Explorer takes a set of gene sequences and searches for locally overrepresented TF-binding sites (TFBS). The search protocol of the TFM-Explorer utilizes weight matrices from the JASPAR vertebrate database [26]. Our input into the program included 3,000 bp upstream and 300 bp downstream sequences of the gene transcription start sites in the FASTA format. Non-coding RNA genes were excluded from the analysis. The significance (*P*-value ≤ 0.01) of the clusters (regions of the input sequences associated with a factor) was based on a score function, as described by Touzet and Varré [27] and Defrance and Touzet [28].

The obtained list of TFs was analyzed for overrepresented gene ontology (GO) terms using the BiNGO (Biological Networks Gene Ontology tool, [29] plug-in of Cytoscape [30]). In the analysis, we applied the default statistical tests and corrections for multiple testing to retain an overall *P*-value of 0.05. A list of the most promising key TFs related to meat pH was formed based on the biological processes involved (e.g. muscle cell homeostasis) and a literature review. The most likely candidate genes were then identified using the NetworkAnalyzer tool within Cytoscape. Based on TFBS (and consequently, the number of connections in each gene and TF), the most connected genes were determined within each list of genes to form a gene-TF network.

## Results

### Significant SNPs

**Fig. 1:**
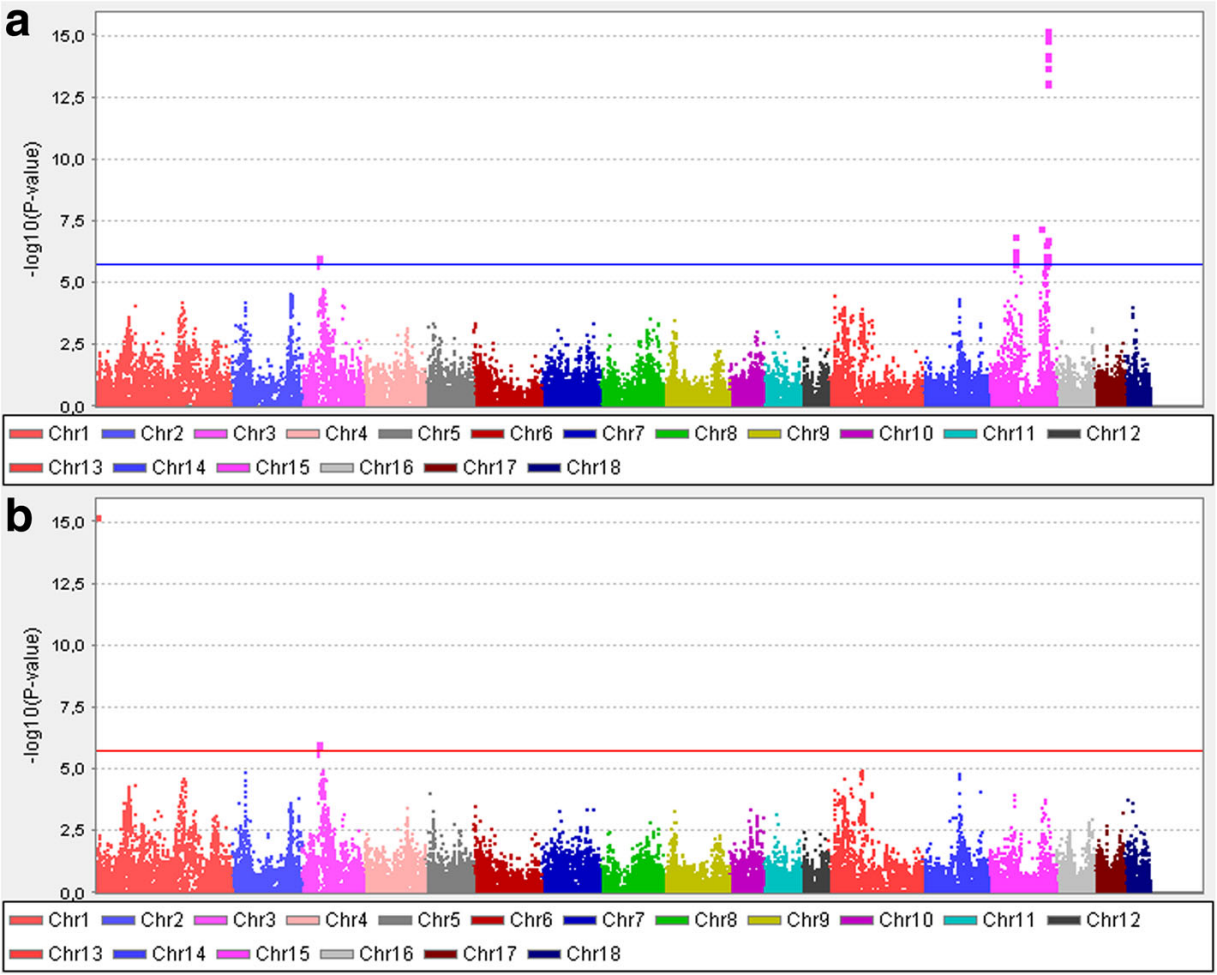
Manhattan plots of *P*-values (-log_10_) from association analysis (**a**) and from conditional association analysis (**b**) of pH measured from loin

**Fig. 2:**
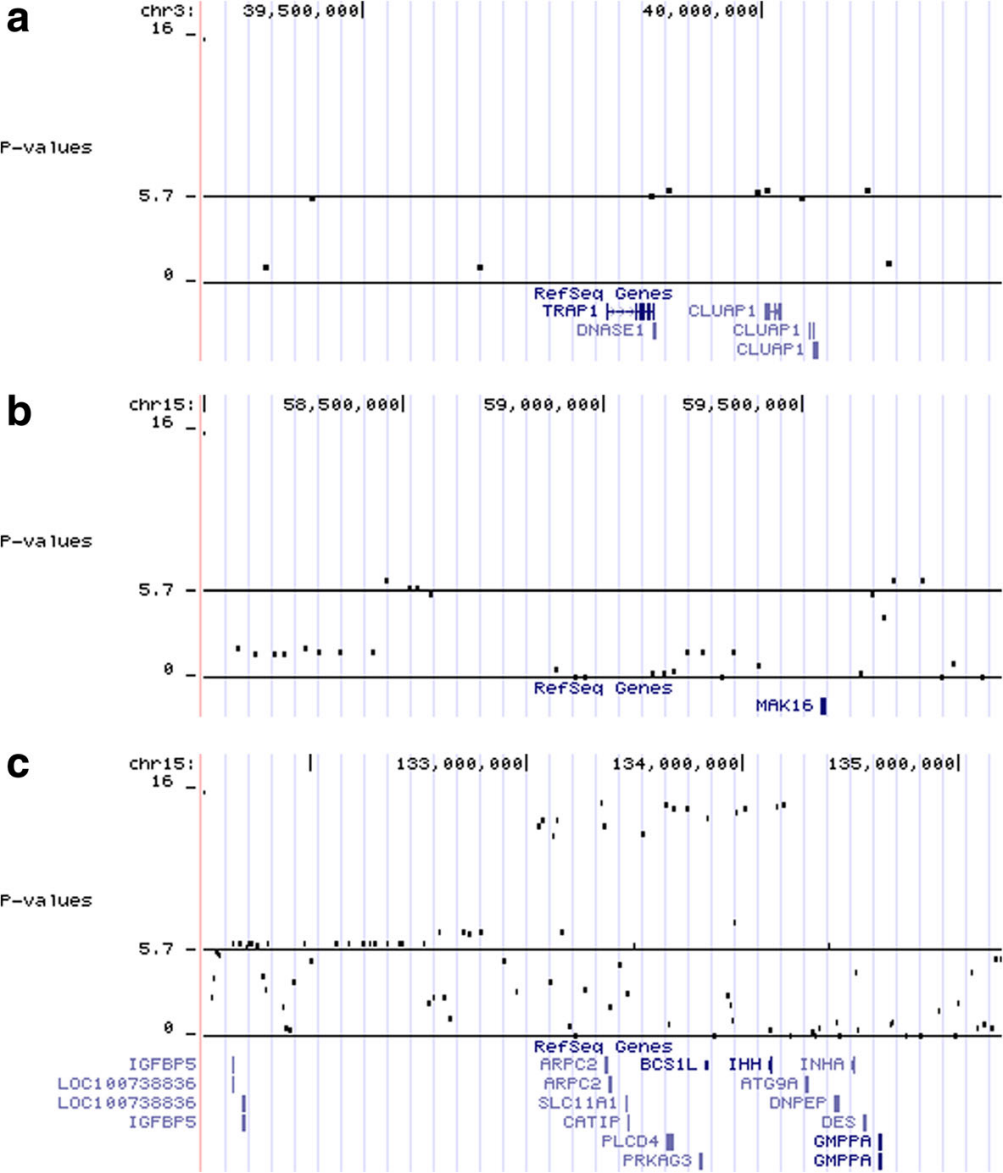
*P*-values (-log_10_-based) of the significant regions. **a**: chromosome 3, **b** and **c**: chromosome 15 against the positions of the validated genes

The Manhattan plots of GWAS are presented in Fig. 1. Three regions were statistically significant: one on chromosome 3 and two on chromosome 15. The allele effects with the standard errors and *P*-values of the significant SNPs are shown in Table 1 and the *P*-values (-log10) against the chromosomal positions with the validated genes from the Reference Sequence database (RefSeq, http://www.ncbi.nlm.nih.gov/refseq/) in Fig. 2.

**Table 1:** Significant (in bold) and close to significant SNPs on chromosomes 3 and 15

SNP	Chr	Position, bp	MAF	CR	Allele	b^a^	SE	n	*P*-value
ALGA0105074	3	39433703	0.50	0.967	G	1.75E-02	3.68E-03	692	2.43E-06
H3GA0009309	3	39858459	0.50	0.999	G	1.75E-02	3.66E-03	702	2.13E-06
**ALGA0018568**	3	39881683	0.50	1.000	A	-1.80E-02	3.63E-03	703	9.14E-07
**M1GA0004302**	3	39992614	0.48	0.952	A	1.81E-02	3.70E-03	674	1.28E-06
**MARC0023922**	3	40003536	0.50	1.000	A	-1.80E-02	3.63E-03	703	9.14E-07
ALGA0018563	3	40047077	0.50	0.978	A	-1.74E-02	3.66E-03	693	2.44E-06
**ASGA0014287**	3	40128902	0.50	0.995	A	-1.80E-02	3.63E-03	701	8.87E-07
**ALGA0112278**	15	58515874	0.06	1.000	G	-3.61E-02	7.48E-03	703	1.70E-06
**ASGA0106157**	15	58532159	0.06	1.000	A	-3.61E-02	7.48E-03	703	1.70E-06
MARC0098560	15	58565300	0.06	0.994	A	-3.51E-02	7.52E-03	694	3.65E-06
ASGA0069641	15	59674662	0.13	1.000	C	-2.62E-02	5.58E-03	703	3.30E-06
**H3GA0044376**	15	59725649	0.10	1.000	G	-3.08E-02	6.07E-03	703	4.91E-07
**ALGA0085452**	15	59799712	0.10	1.000	G	-3.08E-02	6.07E-03	703	4.91E-07
**ASGA0069650**	15	60417583	0.11	1.000	G	-3.12E-02	5.83E-03	703	1.16E-07
**ASGA0069653**	15	60473765	0.06	0.988	A	-3.72E-02	7.54E-03	694	1.04E-06
**ALGA0106581**	15	118661914	0.12	1.000	G	-2.97E-02	5.43E-03	703	6.07E-08
**MARC0105925**	15	129430060	0.32	1.000	G	2.22E-02	4.27E-03	703	2.71E-07
**MARC0012403**	15	130228158	0.35	1.000	A	1.90E-02	3.97E-03	703	1.93E-06
**H3GA0044925**	15	131637011	0.18	0.999	A	2.39E-02	4.79E-03	703	7.56E-07
**ALGA0087027**	15	131665582	0.18	1.000	G	-2.39E-02	4.79E-03	703	7.56E-07
**ALGA0087009**	15	131697805	0.19	1.000	G	-2.28E-02	4.72E-03	703	1.61E-06
**ALGA0087013**	15	131713164	0.18	1.000	A	2.39E-02	4.79E-03	703	7.56E-07
**ASGA0070510**	15	131722929	0.18	1.000	A	2.39E-02	4.79E-03	703	7.56E-07
**ASGA0070514**	15	131745271	0.17	0.957	G	-2.36E-02	4.84E-03	679	1.27E-06
**ALGA0087030**	15	131795839	0.16	1.000	G	-2.88E-02	5.78E-03	488	8.87E-07
**INRA0050208**	15	131966813	0.18	0.999	G	-2.39E-02	4.79E-03	703	7.56E-07
**ASGA0070535**	15	132116033	0.18	1.000	A	2.39E-02	4.79E-03	703	7.56E-07
**ASGA0070533**	15	132171976	0.18	1.000	G	-2.39E-02	4.79E-03	703	7.56E-07
**INRA0050231**	15	132240930	0.18	1.000	A	2.39E-02	4.79E-03	703	7.56E-07
**ASGA0070538**	15	132271433	0.18	1.000	G	2.39E-02	4.79E-03	703	7.56E-07
**H3GA0044934**	15	132293605	0.18	1.000	C	-2.39E-02	4.79E-03	703	7.56E-07
**INRA0050226**	15	132352056	0.18	1.000	G	-2.39E-02	4.79E-03	703	7.56E-07
**ALGA0123666**	15	132411519	0.18	1.000	A	2.39E-02	4.79E-03	703	7.56E-07
**ASGA0101786**	15	132418651	0.18	1.000	G	-2.39E-02	4.79E-03	703	7.56E-07
**ASGA0070540**	15	132520074	0.18	1.000	A	2.39E-02	4.79E-03	703	7.56E-07
**ASGA0070545**	15	132590789	0.17	0.999	A	2.54E-02	4.79E-03	702	1.60E-07
**ASGA0070549**	15	132703653	0.17	0.996	A	2.54E-02	4.81E-03	700	1.70E-07
**ALGA0087060**	15	132730376	0.18	0.989	A	2.55E-02	4.85E-03	695	1.92E-07
**ALGA0087067**	15	132782065	0.18	1.000	A	2.54E-02	4.79E-03	703	1.59E-07
**ASGA0070571**	15	133052815	0.12	1.000	G	-4.11E-02	5.25E-03	703	1.89E-14
**ASGA0070560**	15	133072097	0.13	1.000	A	-3.95E-02	4.98E-03	703	8.24E-15
**H3GA0044951**	15	133118381	0.14	0.999	G	-3.72E-02	4.89E-03	702	9.11E-14
**ASGA0070582**	15	133138277	0.13	1.000	G	-3.95E-02	4.98E-03	703	8.24E-15
**ASGA0070586**	15	133160977	0.40	0.995	G	-2.05E-02	3.87E-03	701	1.65E-07
**ALGA0087116**	15	133342361	0.14	0.999	A	-4.10E-02	4.95E-03	702	6.00E-16
**ALGA0087118**	15	133355327	0.12	0.994	G	-4.12E-02	5.26E-03	700	1.80E-14
**ASGA0070623**	15	133493709	0.38	0.999	G	1.88E-02	3.82E-03	702	1.10E-06
**DRGA0015508**	15	133534807	0.11	0.970	A	-4.14E-02	5.41E-03	680	7.34E-14
**ASGA0070634**	15	133640599	0.13	0.999	A	4.22E-02	5.13E-03	702	8.78E-16
**ASGA0070625**	15	133677385	0.13	1.000	A	-4.17E-02	5.11E-03	703	1.52E-15
**MARC0083357**	15	133738342	0.13	1.000	C	-4.17E-02	5.11E-03	703	1.52E-15
**DBUN0002708**	15	133836471	0.13	0.957	A	4.12E-02	5.16E-03	689	5.70E-15
**MARC0039273**	15	133964455	0.13	1.000	A	-4.17E-02	5.11E-03	703	1.52E-15
**ASGA0070646**	15	133970166	0.12	0.988	A	-4.14E-02	5.18E-03	694	5.59E-15
**DIAS0002965**	15	134006845	0.13	1.000	G	-4.17E-02	5.11E-03	703	1.52E-15
**ASGA0070665**	15	134156879	0.12	0.998	A	-4.31E-02	5.26E-03	701	1.29E-15
**ASGA0070668**	15	134189442	0.12	1.000	G	-4.32E-02	5.25E-03	703	8.21E-16
**MARC0009333**	15	134397712	0.28	0.999	A	-2.02E-02	4.13E-03	702	1.33E-06

^a^Effect of the minor allele, given in the column “Allele”

Chromosome (Chr), Minor allele frequency (MAF), Call rate (CR)

The statistically significant region on chromosome 3 was located at around 39.9 Mb–40.1 Mb and had four significant SNPs with an average allele effect of 0.018 pH units (SE = 0.004), corresponding to 0.4 SD of the total additive genetic effect and approximately 8% of the additive genetic variance (σ_a_ = 0.045 used in national breeding value evaluation for loin pH). In this region, the frequencies of unfavorable and favorable alleles were equal (MAF = 0.5). This region harbors three validated genes: TNF receptor-associated protein 1 (*TRAP1*), deoxyribonuclease I (*DNASE1*), and clusterin-associated protein 1 (*CLUAP1*).

The first region on chromosome 15 (58.5 Mb–60.5 Mb) had six statistically significant SNPs. The allele frequency of the minor allele of the best SNP (ASGA0069650) was 0.11 and the SNP effect was -0.031 pH units (SE = 0.006), corresponding to 0.7 SD of the total additive genetic effect and approximately 9% of the additive genetic variance. Only one gene, MAK16 homolog (*MAK16*), has been validated in this region (Fig. 2).

The second region on chromosome 15 (approximately 130 Mb–134 Mb) included *PRKAG3*, the gene reported earlier using partly the same data as here [12, 31]. The region with highly significant *P*-values contains several other genes besides *PRKAG3*. The allele effect of the most significant SNP (ALGA0087116) was 0.041 pH units (SE = 0.005), corresponding to 0.9 SD of the total additive genetic effect and approximately 20% of the additive genetic variance. Also, a single significant SNP (ALGA0106581) was observed 10 Mb from this region.

**Fig. 3:**
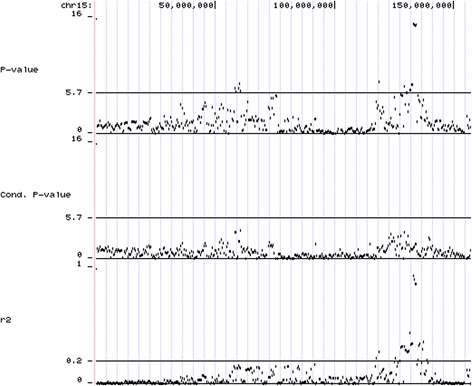
*P*-values (-log_10_-based) from the original and from the conditional association analysis and linkage disequilibrium with MARC0083357 measured as squared correlation (r2) for SNPs on chromosome 15

Because the SNPs at the *PRKAG3* region had far stronger association with pH than the other SNPs on chromosome 15 or on other chromosomes, MARC0083357 was included as a covariate in the model, and GWAS was repeated. MARC0083357 was chosen because it was in complete LD with the most significant SNP ALGA87116, is located very close to *PRKAG3* (62 kb from *PRKAG3*), and had a call rate of 1.0. The conditional GWAS revealed the same significant SNPs on chromosome 3 as the original GWAS. However, the significance of the SNPs in the first region on chromosome 15 (58.5 Mb–60.5 Mb) in the conditional analysis differed from the original GWAS depending on the LD between the SNPs and MARC0083357; e.g., the *P*-value for H3GA0044376 in the conditional GWAS was only 0.017 whereas the original *P*-value was 4.910E-07 (*r* ^2^ with MARC0083357 was 0.16). This reflects the long-distance LD found between MARC0083357 and several SNPs in the region 50 Mb–100 Mb (Fig. 3). However, some of the SNPs, e.g. ASGA0069650 (*r* ^2^ = 0.03), were segregating independently from MARC0083357, and the association with meat pH in the conditional GWAS for these SNPs was still relatively significant (Table 2). Additionally, several SNPs in the vicinity of *PRKAG3* gave relatively small *P*-values, despite the fact that one of the most significant SNPs in that region was included in the model simultaneously (Table 2).

**Table 2:** SNPs on chromosome 15 having a *P*-value less than 1.0E-04 from the conditional GWAS where MARC0083357 was included as a covariate in the model

					Original GWAS		Conditional GWAS			
SNP	Position	MAF	CR	A	b^1^	*P*-value^1^	b^2^	*P*-value^2^	b^1^	*P*-value^1^
ALGA0112278	58515874	0.06	1.00	G	-3.61E-02	1.70E-06	-3.86E-02	1.54E-13	-2.73E-02	1.81E-04
ASGA0106157	58532159	0.06	1.00	A	-3.61E-02	1.70E-06	-3.86E-02	1.54E-13	-2.73E-02	1.81E-04
MARC0098560	58565300	0.06	0.99	A	-3.51E-02	3.65E-06	-3.82E-02	2.79E-13	-2.63E-02	3.43E-04
ASGA0069653	60473765	0.06	0.99	A	-3.72E-02	1.04E-06	-3.87E-02	3.96E-13	-2.85E-02	1.10E-04
ASGA0070395	123808149	0.34	1.00	A	7.93E-03	4.49E-02	-4.49E-02	1.97E-17	1.35E-02	3.76E-04
DRGA0015455	126739060	0.42	0.98	C	-4.74E-03	2.31E-01	-4.66E-02	3.96E-17	-1.29E-02	8.94E-04
ASGA0070433	127982302	0.34	1.00	A	-7.91E-03	4.79E-02	-4.47E-02	2.86E-17	-1.32E-02	5.80E-04
ALGA0086908	129397699	0.49	1.00	G	3.71E-03	3.36E-01	-4.79E-02	3.29E-18	1.42E-02	2.29E-04
MARC0099288	129429610	0.49	0.99	G	4.04E-03	2.95E-01	-4.76E-02	6.73E-18	1.44E-02	1.85E-04
MARC0105925	129430060	0.32	1.00	G	2.22E-02	2.71E-07	-3.77E-02	8.58E-13	1.56E-02	2.01E-04
ALGA0086957	130765546	0.42	0.99	C	3.04E-03	4.12E-01	-4.67E-02	1.74E-17	1.26E-02	6.32E-04

^1^Effect and *P*-value of the minor allele given in the column A

^2^Effect and *P*-value of the minor allele of MARC0083357

### Haplotype analysis

Full LD was obtained for all significant SNPs and very strong LD (*r* ^2^ > 0.97) between all other SNPs in the region on chromosome 3, forming four haplotypes (Table 3). The association between the haplotypes (coded as 0: animal does not carry the haplotype, 1: animal carries one copy of the haplotype, and 2: animal carries two copies of the haplotype) and pH confirmed the positive effect of haplotype 1 on loin pH (Table 3). Compared to the rest of the haplotypes, the effect of haplotype 1 on loin pH was 0.018 (SE = 0.004), the same as in the single-SNP analysis.

**Table 3:** Haplotypes in the 39.4 Mb–40.1 Mb region on chromosome 3 and their association with pH

Haplotype	Nucleotides^1^	Frequency	b^2^	SE	*P*-value
1	AGAACAAA	0.50	1.76E-02	3.60E-03	1.26E-06
2	GGGGAGGG	0.45	-1.53E-02	3.73E-03	4.58E-05
3	GAGGAGGG	0.05	-1.39E-02	8.23E-03	NS^3^
4	AGAGAGGG	<0.01	-1.88E-02	2.01E-02	NS

^1^The SNPs are: ALGA0105074, ALGA0114510, H3GA0009309, ALGA0018568, M1GA0004302, MARC0023922, ALGA0018563, ASGA0014287. ^2^Effect of the haplotype. ^3^NS = not significant

**Fig. 4:**
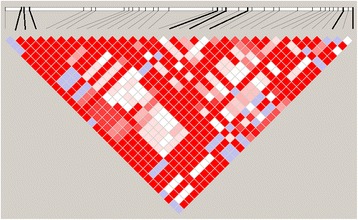
Linkage disequilibrium plots of the region 58.5 Mb–60.5 Mb on chromosome 15. SNPs presented in Table 2 are marked with *bold lines*

The linkage disequilibrium structure (D’) of the first region on chromosome 15 (58.5 Mb–60.5 Mb) is presented in Fig. 4. The significant region from the original GWAS is approximately 2 Mb long and includes over 30 SNPs that passed the quality check. Even though the LD structure presented as D’ values (Fig. 4) is quite strong throughout the region, there are still 16 different haplotypes present. Haplotype 6, with very low frequency (0.05), showed strong association with loin pH: a haplotype effect of -0.036 pH units with SE = 0.008 and *P*-value = 6.6E-06 (Table 4). Also haplotype 7 showed a similar haplotype effect (-0.021, SE = 0.009) but with a higher *P*-value of 0.017. All other haplotypes gave a *P*-value > 0.05. Haplotypes 6 and 7 only have 30% of the alleles in common, but share three significant SNPs: H3GA0044376, ALGA0085452, and ASGA0069650 (see Table 2). Considering these two haplotypes together against all other haplotypes gave a *P*-value of 4.95E-07.

**Table 4:** Haplotypes in the 58.5 Mb–60.5 Mb region on chromosome 15 and their association (b) with pH

Haplotype	Nucleotides^1^	Frequency^2^	b	SE	*P*-value
1	GAGGGAAAGGGGAAGAACAAAGGAGAGGGAGAAAG	0.25	8.59E-03	4.59E-03	NS^3^
2	GAGGGAAGAAGGAAAGACAAAAGAGAAAGAGAAAG	0.22	4.30E-03	4.36E-03	NS
3	GAGGAGGGAGGGAAGGACAAGAAAGAAGGAGAAAG	0.16	1.07E-03	5.38E-03	NS
4	GAGGAGGGAGGGGAAGACAAGAAGAGGAGAGAAAG	0.13	5.58E-03	5.42E-03	NS
5	GAGGGAAAGGGGGAAGACAAGAAGAGGAGAGAAAG	0.08	-5.27E-03	6.26E-03	NS
6	GGAAGAAAGGGGAAGACGGGAGGAGAAAGGAAGAA	0.05	-3.63E-02	8.00E-03	6.69E-06
7	AAGGGGGGAGAAGGGGCCGGGGGAAGGGAAGAGAG	0.05	-2.15E-02	8.98E-03	0.02
8	GAGGAGGGAAGGAAGGCGAAGAGAGAAAGAGAAAG	0.03	-2.25E-03	1.43E-02	NS
9	GAGGGAAAGGGGGAAGACAAGAGGGAAGGGAGACG	0.03	2.15E-02	1.12E-02	NS

^1^The SNPs are: INRA0049382, ALGA0112278, ASGA0106157, MARC0098560, DRGA0015140, DRGA0015141, ALGA0085422, MARC0044358, ALGA0085430, ALGA0085436, ALGA0085439, INRA0049399, ALGA0085441, ALGA0085442, MARC0043488, DRGA0015145, ASGA0069641, ALGA0085445, H3GA0044376, ALGA0085452, MARC0000855, ASGA0069644, ALGA0085462, H3GA0044381, INRA0049414, H3GA0044383, MARC0071088, ALGA0085464, ALGA0085465, MARC0073466, INRA0049417, DRGA0015149, ASGA0069650, ALGA0085471, ASGA0069653. ^2^Only haplotypes with a frequency greater than 1% are presented. ^3^NS = not significant

We also attempted a haplotype analysis for the second region of chromosome 15, but no further information was attained beyond the results presented for single SNPs. The reason for this is the length of the significant region, which created a very large number of possible haplotypes depending on the way haplotype blocks were defined.

### Post-GWAS analysis

**Fig. 5:**
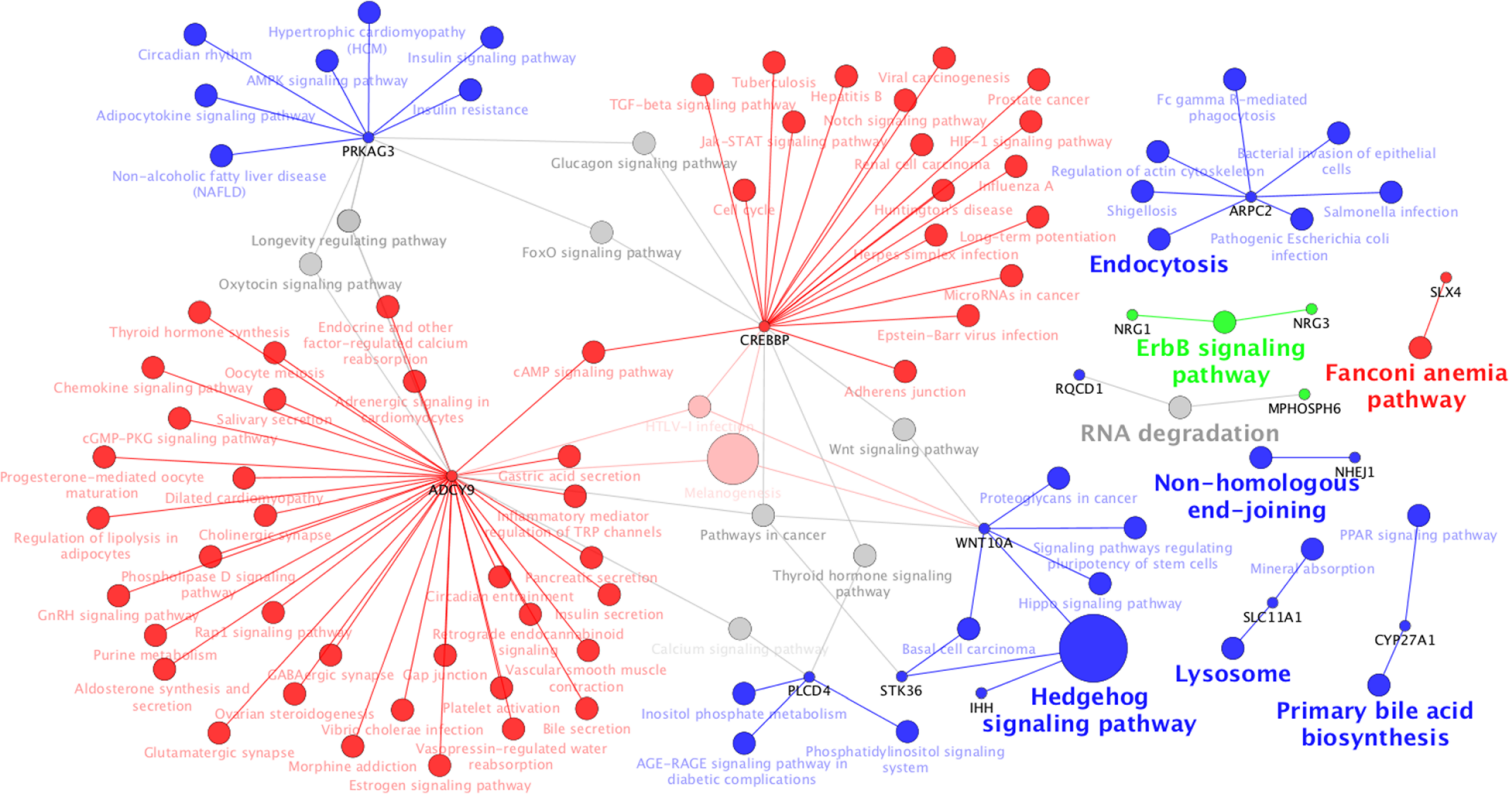
Main functional group networks with pathway terms and genes as nodes. *Red nodes* represent pathways associated with genes on chromosome 3 (39.4 Mb–40.1 Mb); *green* and *blue nodes* are pathways associated with genes on chromosome 15 (58.5 Mb–60.5 Mb and 133.3 Mb–134.2 Mb, respectively). *Pink* and *gray nodes* are pathways shared between the regions. The most enriched terms per group are shown in *bold* according to enrichment significance from the ClueGO Cytoscape plug-in. Node size corresponds to enrichment significance. Nodes named in *black* are the observed genes

Based on the database annotation, a total of 56 genes were located next to the significant SNPs or regions identified in GWAS (Table 5). These genes were found to form main functional networks (Fig. 5). The genes on chromosome 3 (e.g. *CREBBP* and *ADCY9*) and on chromosome 15 (e.g. *PRKAG3, IHH, WNT10A, STK36*, and *PLCD4* at 133.3 Mb–134.2 Mb) share statistically the most significant pathway, the Hedgehog signaling pathway. Other highlighted network pathways include Endocytosis, ErbB signaling, Faconiani anemia, RNA degradation, Non-homologous end-joining, Lysosome, and Primary bile acid biosynthesis.

**Table 5:** List of genes that locate inside significant regions or are in the proximity of the significant SNPs not included in any of the three regions

SNP^1^	Chr^2^	Position	Region	Genes	Distance (kb)^3^
*ALGA0105074*	3	39433703	Region 1	*NLRC3, TRAP1, SLX4, LOC100738967/CREBBP, LOC100738923/CREBBP, LOC100738884/ADCY9, DNASE1, CREBBP, CLUAP1* and *C3H16orf90*	Inside
*H3GA0009309*	3	39858459			
ALGA0018568	3	39881683			
M1GA0004302	3	39992614			
MARC0023922	3	40003536			
*ALGA0018563*	3	40047077			
ASGA0014287	3	40128902			
ALGA0112278	15	58515874	Region 2	*TTI2, RNF122, MAK16, LOC102164955/NRG1, LOC102164569/NRG1, LOC100739280/NRG1, LOC100739161/MPHOSPH6, LOC100620584/NRG3* and *DUSP26*	Inside
ASGA0106157	15	58532159			
*MARC0098560*	15	58565300			
*ASGA0069641*	15	59674662			
H3GA0044376	15	59725649			
ALGA0085452	15	59799712			
ASGA0069650	15	60417583			
ASGA0069653	15	60473765			
ALGA0106581	15	118661914	-	-	-
MARC0105925	15	129430060	-	-	-
MARC0012403	15	130228158	-	-	-
H3GA0044925	15	131637011	-	*LOC100738836/IGFBP5*	2.091
ALGA0087027	15	131665582	-	*IGFBP5*	16.680
ALGA0087009	15	131697805	-	*IGFBP5*	13.200
ALGA0087013	15	131713164	-	-	-
ASGA0070510	15	131722929	-	-	-
ASGA0070514	15	131745271	-	-	-
ALGA0087030	15	131795839	-	*TNP1*	4.532
INRA0050208	15	131966813	-	-	-
ASGA0070535	15	132116033	-	-	-
ASGA0070533	15	132171976	-	-	-
INRA0050231	15	132240930	-	-	-
ASGA0070538	15	132271433	-	-	-
H3GA0044934	15	132293605	-	-	-
INRA0050226	15	132352056	-	-	-
ALGA0123666	15	132411519	-	-	-
ASGA0101786	15	132418651	-	-	-
ASGA0070540	15	132520074	-	-	-
ASGA0070545	15	132590789	-	-	-
ASGA0070549	15	132703653	-	-	-
ALGA0087060	15	132730376	-	-	-
ALGA0087067	15	132782065	-	-	-
ASGA0070571	15	133052815	-	*TNS1*	Inside
				*LOC100514326/tensin-1-like*	Inside
ASGA0070560	15	133072097	-	*TNS1*	Inside
				*LOC100514326/tensin-1-like*	Inside
H3GA0044951	15	133118381	-	*LOC102160493/tensin-1-like*	Inside
				*LOC100514326/tensin-1-like*	Inside
				*TNS1*	7.489
ASGA0070582	15	133138277	-	*LOC102160493/tensin-1-like*	Inside
				*LOC100514326/tensin-1-like*	Inside
ASGA0070586	15	133160977	-	*LOC102160493/tensin-1-like*	10.500
				*LOC100514326/tensin-1-like*	Inside
ALGA0087116	15	133342361	Region 3	*ZNF142, USP37, TTLL4, STK36, SLC11A1, RQCD1, RNF25, PRKAG3, PLCD4, NHEJ1, LOC102163340/CCDC108, LOC102163099/CCDC108, LOC102162367/tubulin polyglutamylase TTLL4-like, LOC102162096/ZNF142, LOC102161979/mitochondrial chaperone BCS1, LOC102161492/VIL1, PNKD, LOC100739763/PRKAG3, AAMP, GPBAR1, TMBIM1, LOC100739428, WNT10A, VIL1, IHH, CYP27A1, CRYBA2, CDK5R2, CCDC108, C15H2orf62* and *ARPC2*	Inside
ALGA0087118	15	133355327			
ASGA0070623	15	133493709			
DRGA0015508	15	133534807			
ASGA0070634	15	133640599			
ASGA0070625	15	133677385			
MARC0083357	15	133738342			
DBUN0002708	15	133836471			
MARC0039273	15	133964455			
ASGA0070646	15	133970166			
DIAS0002965	15	134006845			
ASGA0070665	15	134156879			
ASGA0070668	15	134189442			
MARC0009333	15	134397712	-	-	-

^1^Non-significant SNPs that included in the three regions are marked in italics. ^2^CHR: chromosome. ^3^Distance from the closest significant SNP

**Fig. 6:**
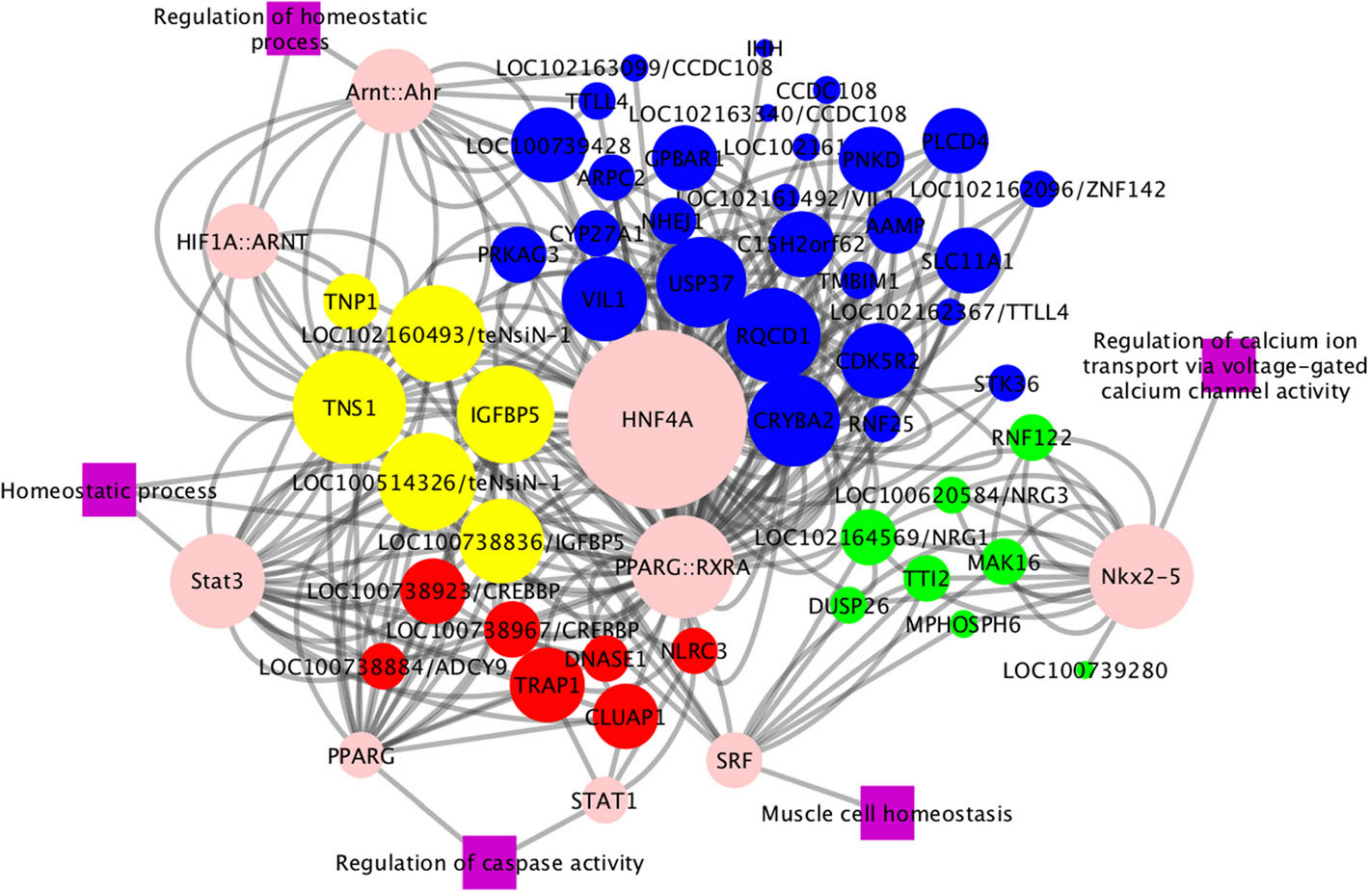
Gene-transcription factor (TF) network. Transcription factors (*pink nodes*) and genes overlapping with relevant SNPs or regions for pH (*yellow nodes* are genes observed to be associated with single SNP group; r*ed nodes* are genes observed in chromosome 3 region (39.4 Mb–40.1 Mb); *green nodes* are genes observed in the first region on chromosome 15 (58.5 Mb–60.4 Mb); *blue nodes* are genes observed in the second region on chromosome 15 (133.3 Mb–134.2 Mb). The node size corresponds to the network analysis (Cytoscape) score, where *bigger nodes* represent higher edges density associated with the number of TF-binding sites. *Purple square nodes* are biological processes (GO terms) associated with muscle pH

In addition, the four sets of genes in the significant chromosomal regions were used as input for the TFM-Explorer. Twenty-five TFs were identified through this analysis in each set. Based on the biological processes overrepresented in the BiNGO as well as on a literature review related to meat pH, we selected eight key TFs (Table 6) to construct a combined gene-TF network (Fig. 6). This network highlights the most connected genes within each set (e.g. *TNS1, IGFBP5, VIL1, USP37, RQCD1, CRYBA2, PRKAG3, MAK16, NRG1, TRAP1, CLUAP1*, and *CREBBP*) and provides an overview of shared TFs and genes across the single significant SNPs and regions on chromosomes 3 and 15.

**Table 6:** Main transcription factors (TF) associated with genes overlapping with significant SNPs or regions and their biological process and literature evidences related to meat pH

TF	Group	Biological Process (GO)	Literature evidence
*PPARG*	Single SNPs and Region 1^1^	Regulation of caspase activity	Lipid and glucose homeostasis [47]
*SRF*	Region 2	Muscle cell homeostasis	Actin cytoskeleton and contractile homeostasis [59]
*Stat3*	Single SNPs and Region 1	Homeostatic process	Glucose Homeostasis [60]
*PPARG::RXRA*	Single SNPs and Regions 1 and 3	Homeostatic process	Lipid and glucose homeostasis [47]
*Arnt::Ahr*	Single SNPs and Region 3	Regulation of homeostatic process	Cellular homeostasis [61]
*STAT1*	Region 1	Regulation of caspase activity	Cellular homeostasis [62]
*Nkx2-5*	Region 2	Regulation of calcium ion transport via voltage-gated calcium channel activity	Homeostasis [49]
*HNF4A*	Single SNPs and Region 3	Homeostatic process	Lipid homeostasis [46]

^1^Region 1: chromosome 3 (39,9 Mb–40.1 Mb); Region 2: chromosome 15 (58.5 Mb–60.5 Mb); Region 3: chromosome 15 (133.3 Mb–134.2 Mb)

## Discussion

This article presents the results of GWAS and post-GWAS of pH measured from loin using the Finnish Yorkshire pig breed. A previous GWAS with 470 animals which were also included in the current study revealed two statistically significant (*P*-value < 2.0E-06) chromosomal regions: one on chromosome 2 and one on chromosome 15 (*PRKAG3* region) [12]. As in the previous study, the region around *PRKAG3* also gave the smallest *P*-value in this study. The SNPs on chromosome 2 found significant in the previous study [12] now reached a *P*-value of 5.77E-05 (ASGA0009838) that is still very close to significance and can be considered as “suggestive” finding. In the previous study, the best SNP on chromosome 3 (ASGA0014287) had a *P*-value of 5.89E-05. The best SNP on chromosome 15 at 58.5 Mb–60.5 Mb (ASGA0069650) also had an almost significant *P*-value (5.75E-06) in the previous study. Thus, increasing the number of observations from 470 to 703 changed the results somewhat. Results based on larger data are obviously more reliable than those based on smaller datasets.

Long-distance LD was observed between the SNPs in the *PRKAG3* region and the other SNPs on chromosome 15. Including MARC0083357 in the model reduced the effect and significance of all SNPs on chromosome 15. Thus the significance observed in the first region on chromosome 15 may be “reflection” of the PRKAG3 region due to LD. However, the question why LD between these regions is so strong remains unsolved. Altogether, the mechanism on chromosome 15 that reduces pH in muscle post mortem is currently not yet fully understood. Beyond the original 200Q allele of *PRKAG3*, the functional variations could be due to other genes or to interaction between genes and transcription factors. Several studies have given evidence that I199V is highly associated with loin pH [32–35]. Also Rubio et al. [14] considered PRKAG3 as the best candidate in the region 132 Mb–135 Mb of chromosome 15 (the same region as detected in our study). However, based on a previous study by Uimari and Sironen [31], I199V is not directly associated with loin pH in the Finnish Yorkshire population; instead, the haplotype g.-157C–g.-58A–24E–199I is. Also Ryan et al. [36] reported a positive association of g.-157C with *PRKAG3* expression and meat quality, and Zhang et al. [15] proposed that other genes besides *PRKAG3* could be responsible for the association found in this region.

### Post-GWAS

All the detected significant regions contain several genes. To find the most promising candidate genes within these regions and to understand the possible relationship between the candidate genes, a post-GWAS analysis was performed. Hedgehog signaling was the most significantly enriched pathway in the main functional group network (Fig. 5) comprising seven genes (*PRKAG3*, *ADCY9*, *CREBBP*, *PLCD4*, *IHH*, *WNT10A*, and *STK36*). The hedgehog signaling pathway has been cited as a key factor in the regulation of human adult tissue homeostasis and repair. It acts via multiple different routes to regulate distinct cellular outcomes, including the maintenance of plasticity [37]. Among the genes present in this pathway, *ADCY9* is a type 9 adenylyl cyclase, which is involved in the main pathways promoting muscle relaxation by a cAMP (cyclic AMP) messenger [38]. *PLCD4* is connected in the network through the calcium signaling and thyroid hormone signaling pathways. The phospholipase C enzyme promotes smooth muscle relaxation [39] and generates a second messenger IP3 that controls many cellular processes by inducing intracellular calcium mobilization [40].

The significant region of chromosome 3 contains two genes (*CREBBP* and *ADCY9*) involved in cyclic cAMP signaling. cAMP signaling mediates the effects of metabolism-controlling hormones, such as glucagon and epinephrine, and regulates energy homeostasis in multiple tissues [41]. cAMP is generated from ATP by adenylate cyclase enzymes, one of which is encoded by *ADCY9* (adenylate cyclase 9) [42]. *ADCY9* displays high expression in skeletal muscle and responds to beta-adrenergic receptor activation [43], which modulates Ca^2+^ release from the sarcoplasmic reticulum. Ca^2+^ release is a major contributor to pork meat quality, as is the case in the mutation R615C found in the pale, soft, and exudative (PSE) meat-related ryanodine receptor gene *RYR1* [6]. cAMP activates protein kinase A (PKA), which controls metabolism either directly or through gene expression by phosphorylating the transcription factor CREB (cAMP-response element-binding protein), and consequently leading to the recruitment of its cofactor CBP (CREB binding protein) encoded by *CREBBP* [44]. Together, CREB and CBP regulate a plethora of metabolic target genes involved in glucose metabolism [41], which can potentially impact meat acidification. In addition to transcriptional regulation, PKA-mediated phosphorylation activates phosphorylase kinase, which stimulates glycogen phosphorylase to active glycogen release (glycogenolysis), which, in turn, is directly reflected on lactate production [41]. Moreover, PKA phosphorylates *RYR1* and consequently regulates calcium release in skeletal muscle [45], providing another putative mechanism for the impact of *ADCY9* on meat quality.

Each of the four sets of genes was also used to explore the promoter regions for enriched TFs, of which we selected the most relevant ones for meat pH to generate gene-TF networks. These networks were merged, enabling the visualization of the genes and the TFs that are common between genes in the different regions. The most connected TF (linked with a high number of genes) in this network is *HNF4A*, followed by *PPARG* and *Nkx2-5. HNF4A* encodes to hepatocyte nuclear factor 4α, which is essential to control the basal expression of genes involved in lipid metabolism and is indispensable for maintaining normal lipid homeostasis [46]. *PPARG* is the most studied isoform of the nuclear receptor superfamily, and performs an important role in regulating lipid and glucose homeostasis, in adipocytes differentiation, and in fatty acid storage [47]. Moreover, the polymorphism of this gene has been associated with meat quality traits in cattle [48]. The third most connected TF is *Nkx2-5*, which is a homeobox transcription factor known to be required for homeostasis of cardiac myocytes [49]. Briggs et al. [50] observed that *Nkx2-5* knockout mice presented a reduced expression of ryanodine receptor 2 (*RYR2*), through which calcium is released from the sarcoplasmic reticulum. The corresponding skeletal muscle-related ryanodine receptor gene is *RYR1*. The most relevant biological processes of these TFs that affect meat pH are the homeostatic process (*HNF4A* and *PPARG*) and the regulation of calcium ion transport via voltage-gated calcium channel activity (*Nkx2-5*).

In the gene-TF network, *HNF4A* and *PPARG* are mainly connected with the genes on chromosome 3 and in the second region on chromosome 15, while genes identified in the first region of chromosome 15 are mainly connected with *Nkx2-5*. In the significant region on chromosome 3, *TRAP1* is the most connected gene in the gene-TF network. *TRAP1* encodes a mitochondrial chaperone protein that interacts with a calcium-binding protein, sorcin, and is thus involved in intracellular calcium concentration [51] and might also affect muscle pH. Of the genes in the first significant region on chromosome 15, the most connected gene in the gene-TF network is *LOC102164569/NRG1* (pro-neuregulin-1, membrane-bound isoform-like). This gene is a member of the neuregulin growth factor gene family that is involved in the differentiation of embryonic muscle cells [52]. It has also been cited to be related to alterations of intracellular calcium homeostasis in humans [53] and to the marbling trait in Korean Hanwoo cattle [54]. We observed other genes besides *PRKAG3* in the second region on chromosome 15 that are highlighted in the gene-TF network, such as *VIL1*, *USP37*, *RQCD1*, and *CRYBA2*. Among them, *VIL1* has also been associated with meat pH and color in crossbred commercial pigs [15]. The most connected gene a bit further apart (700 kb) from *PRKAG3* is *TNS1* (tensin 1), which belongs to a focal adhesion gene family and interacts with actin filaments [55]. Recently, *TNS1* has been identified as a candidate gene in GWAS for meat pH in Chinese Laiwu pigs [56]. Another gene, *IGFBP-5*, 2 Mb from *PRKAG3,* is a member of the IGF gene families. It has been suggested to be associated with meat quality, especially with pH in pigs [57]. Moreover, the mRNA level of this gene in muscle samples was observed to be significantly lower in Duroc pigs compared to other commercial breeds [58]. *TNS1* and *IGFBP5* are the most connected genes in the whole gene-TF network, and might thus be considered very stronger candidate genes for meat pH.

## Conclusions

Three regions, one on chromosome 3 (39.4 Mb–40.1 Mb) and two on chromosome 15 (58.5 Mb–60.5 Mb and 133.3 Mb–134.2 Mb), were found to be highly associated with meat pH in Finnish Yorkshire. Additionally, several other SNPs up to a distance of 3 Mb from the known meat quality gene *PRKAG3* proved significant. The significant regions harbored several genes, including two genes involved in cAMP signaling: *ADCY9* and *CREBBP*. Based on post-GWAS analysis using functional and TF-gene networks, the most promising candidate genes for meat pH are *ADCY9, CREBBP, TRAP1, NRG1, PRKAG3*, *VIL1, TNS1*, and *IGFBP5*. The key TFs related to these genes are *HNF4A*, *PPARG*, and *Nkx2-5*. To conclude, we succeeded in identifying several genes which, based on SNP association, pathway, and transcription factor analysis, have potential to control muscle cell homeostasis and meat quality. However, functional studies are still needed to warrant the role of each of these genes in pork meat quality.

## Abbreviations

cAMP: Cyclic AMP
CBP: CREB binding protein
CR: Call rate
CREB: cAMP-response element-binding protein
dEBV: Deregressed EBV
EBV: Estimated breeding value
GO: Gene ontology
GWAS: Genome-wide association study
LD: Linkage disequilibrium
MAF: Minor allele frequency
N_e_: Effective population size
PKA: Protein kinase A
PSE: Pale, soft, and exudative
REML: Restricted maximum likelihood
TF: Transcription factor
TFBS: Transcription factor binding site
